# Next generation self-replicating RNA vectors for vaccines and immunotherapies

**DOI:** 10.1038/s41417-022-00435-8

**Published:** 2022-02-22

**Authors:** Parinaz Aliahmad, Shigeki J. Miyake-Stoner, Andrew J. Geall, Nathaniel S. Wang

**Affiliations:** Replicate Bioscience, Inc., San Diego, CA USA

**Keywords:** Genetic vectors, Nucleic-acid therapeutics, Cell delivery, Drug development, RNA vaccines

## Abstract

RNA technology has recently come to the forefront of innovative medicines and is being explored for a wide range of therapies, including prophylactic and therapeutic vaccines, biotherapeutic protein expression and gene therapy. In addition to conventional mRNA platforms now approved for prophylactic SARS-CoV2 vaccines, synthetic self-replicating RNA vaccines are currently being evaluated in the clinic for infectious disease and oncology. The prototypical srRNA vectors in clinical development are derived from alphaviruses, specifically Venezuelan Equine Encephalitis Virus (VEEV). While non-VEEV alphaviral strains have been explored as single cycle viral particles, their use as synthetic vectors largely remains under-utilized in clinical applications. Here we describe the potential commonalities and differences in synthetic alphaviral srRNA vectors in host cell interactions, immunogenicity, cellular delivery, and cargo expression. Thus, unlike the current thinking that VEEV-based srRNA is a one-size-fits-all platform, we argue that a new drug development approach leveraging panels of customizable, synthetic srRNA vectors will be required for clinical success.

## Introduction

Self-replicating RNA (srRNA) as a vaccine and therapeutic modality has been increasingly used for the in situ production of proteins. srRNAs are derived from positive-strand RNA viruses where the structural proteins have been removed and replaced with heterologous genes of interest [1–3]. srRNAs have been successfully derived from flaviviruses, nodamura viruses, nidoviruses, and alphaviruses with therapeutic versions of the technology providing the structural proteins in *trans* to create single cycle viral replicon particles (VRPs). Alphavirus VRPs have been shown to be safe and well tolerated with promising responses in extensive preclinical characterizations and Phase I/IIa clinical trials in both infectious disease and oncology [4] (Table 1). More recently, fully synthetic versions of the technology have been developed, where the viral structural proteins are replaced with a protective coat in the form of a lipid nanoparticle (LNP) or polymers [5]. The first attempts of clinical translation with LNP-formulated synthetic srRNAs demonstrated safety and immunogenicity, with assets advancing to PhII in oncology and PhIII in infectious disease [5] (Table 1). Synthetic srRNAs have several advantages over traditional viral vectors, namely increased safety profile based on the lack of potential for genomic integration or cell transformation, and simplified manufacturing. In addition, lack of a viral shell results in no/lower anti-vector immunity allowing repeated dosing and the ability to encode multiple larger genes of interest, normally limited by the packaging capacity of the viral particle. Furthermore, another safety advantage of srRNA platforms is the reduced efficacious human dose. As srRNA amplify within the host cell, low doses still result in higher and more durable protein expression making them advantaged for biotherapeutics compared to mRNA [6]. Lastly, current clinical srRNA-based vaccines are advantaged compared to mRNA due to their ability to elicit robust cell-mediated immunity, exemplified by strong CD8+ and CD4+ T cell responses critical for efficacious oncology therapeutics.

**Table 1:** Selected self-replicating RNA clinical trials.

Therapeutic area	Name	Antigen/protein	Phase	VRP or srRNA	species	Formulation	ROA	Dose (IU or ug)	Entity	Trial number	Results
Infectious Disease	AVX601	CMV gB, pp65/IE1	I	VRP	VEEV	None	IM	10^7^, 10^8^	AlphaVax	NCT00439803	Well-tolerated; Antibody and polyfunctional antigen-specific T cell responses
	AVX101	HIV Gag	I	VRP	VEEV	None	IM	10^4^-10^8^	AlphaVax	NCT00063778, NCT00097838	Well-tolerated, low immunogenicity
	AVX502	Influenza HA	I/II	VRP	VEEV	None	IM, SC	Not listed	AlphaVax	NCT00440362	Well-tolerated; 86% seroconversion and T cell responses.
	ARCT-021	SARS-CoV-2	III	srRNA	VEEV	LNP	IM	5	Arcturus	Initiation H2-2021	Interim data: >90% seroconversion after a single dose for IgG antibodies
	ARCT-154	SARS-CoV-2	I/II/III	srRNA	VEEV	LNP	IM	5	Arcturus	NCT05012943	No data reported
	LNP-nCoVsaRNA	SARS-CoV-2	I	srRNA	VEEV	LNP	IM	0.1-10	ICL	ISRCTN17072692	Well-tolerated; Neutralizing antibody seroconversion varied from 15-48% depending on dose level.
	CORAL	SARS-CoV-2	I	srRNA	VEEV	LNP	IM	10, 30	Gritstone	NCT04776317	Homologous srRNA regimen and heterologous with ChAd
	BNT162c2	SARS-CoV-2	I/II	srRNA	VEEV	LNP	IM	Not listed	BioNTech	NCT04380701	Single dose
	CoV2 SAM	SARS-CoV-2	I	srRNA	VEEV-SINV	LNP	IM	Not listed	GSK	NCT04758962	No data reported
	GSK4108771A	HSV-2	I	srRNA	VEEV-SINV	LNP	IM	Not listed	GSK	NCT04762511	Terminated
	RG SAM	Rabies	I	srRNA	VEEV-SINV	CNE	IM	Not listed	GSK	NCT04062669	No data reported
Oncology	AVX901	HER2	I	VRP	VEEV	None	IM	4×10^8^	AlphaVax	NCT01526473	Well-tolerated; HER2-specific memory T cell population correlated with improved progression free survival
	AVX701	CEA	I	VRP	VEEV	None	IM	0.4 × 10^8-^4 × 10^8^	AlphaVax	NCT00529984	Well-tolerated; Prolonged survival, T cell responses detected
	PSMA-VRP	PSMA	I	VRP	VEEV	None	IM	0.9 × 10^7^, 0.36 × 10^8^	AlphaVax	PMID: 23246260	Well-tolerated; No clinical benefit due to suboptimal dosing
	LSFV-IL-12	IL-12	I/II	VRP	SFV	liposome	IV or IP	1 × 10^8^, 1 × 10^9^	Regulon	PMID: 12952295	No grade III or IV toxicities
	CYN102	NYESO-1	I	VRP	SINV	None	IV or IP	Not listed	Cynvec	Initiation planned 2021	Not yet initiated
	Vvax001	HPV16 E6 E7	I	VRP	SFV	None	IM	5 × 10^5^-2.5 × 10^8^	ViciniVax B.V	NCT03141463	Well-tolerated; immune responses observed in all participants
	GRT-C901/2	Neoantigens	II	srRNA	VEEV	LNP	IM	Not listed	Gritstone	NCT03639714	ChAd prime + /− srRNA: Interim data: 44% molecular response rate by ctDNA analysis in CRC.
	GRT-C903/4	Shared neoantigens	II	srRNA	VEEV	LNP	IM	Not listed	Gritstone	NCT03953235	ChAd prime + /− srRNA + /− checkpoint inhibitors. Unconfirmed partial response in NSCLC. Doses well-tolerated

Despite these built-in advantages, recent clinical candidates in the infectious disease space reveal the challenges of developing fully synthetic srRNA products. Current clinical srRNA-based SARS-CoV-2 vaccine candidates are dosed an order of magnitude lower versus mRNA platforms. This is due, in part, to dose-limiting toxicities observed from the drug product itself [7, 8]. While numerous clinical studies have shown no inherent toxicity related to the biology of the virally derived RNA itself in both viral particle and fully synthetic forms [9–13], impurities caused by poor manufacturing can lead to non-specific, systemic inflammatory responses that drive dose-dependent adverse events witnessed in srRNA COVID-19 vaccine candidates [5, 14–17]. Additional contributing factors include the specific LNP compositions, which have led to differences in tolerability in RNA vaccine candidates that are otherwise similar in design and use of modified bases [18–20]. Interestingly, adverse event profiles at these low doses were not observed in trials in oncology with srRNA formulated in LNPs [13] (Table 2). The net clinical result of these developmental complexities has been suboptimal seroconversion, poor antibody titers, and dose-limiting toxicities at low doses when compared to infectious disease candidates utilizing modified mRNA. Nevertheless, the ability to elicit both superior T cell responses and antibody titers capable of matching convalescent patients at much lower doses shows the promise of the technology once the design and manufacturing are improved, especially in pandemic settings [14, 15, 21, 22].

**Table 2:** Clinical tolerated doses of RNA-based medicines.

Name	Company	Bases	Formulation	ROA	Target	Highest dose reported with favorable safety profile	Ref
mRNA-1893	Moderna	Modified	Internal LNP	Intramuscular	Zika	30 µg (interim reporting, 100 and 250 µg doses pending)	[85]
VAL-506440; VAL-339851	Moderna	Modified	Internal LNP	Intramuscular, Intradermal	Influenza	100 µg (IM), 50 µg (ID)	[86, 87]
mRNA-1273	Moderna	Modified	Internal LNP	Intramuscular	SARS-CoV-2	100 µg	[7, 87, 88]
BNT162b2	Pfizer/BioNTech	Modified	Acuitas LNP	Intramuscular	SARS-CoV-2	30 µg	[89, 90]
CVnCoV	CureVac	Unmodified	Acuitas LNP	Intramuscular	SARS-CoV-2	12 µg	[91]
ARCT-21	Arcturus	Unmodified	Internal LNP	Intramuscular	SARS-CoV-2	7.5 µg	[14]
LNP-nCoVsaRNA	ICL/VaxEquity	Unmodified	Internal LNP	Intramuscular	SARS-CoV-2	10 µg	[15]
CORAL (BOOST)	Gritstone	Unmodified	Genevant LNP	Intramuscular	SARS-CoV-2	10 µg (interim reporting, 30 µg dose pending)	[92]
MRT5500	Translate Bio	Unmodified	Internal LNP	Intramuscular	SARS-CoV-2	45 µg (no DLTs reported)	[93, 94]
CV7202	CureVac	Unmodified	Acuitas LNP	Intramuscular	Rabies	2 µg	[95]
SLATE/GRANITE	Gritstone	Unmodified	Genevant LNP	Intramuscular	Neoantigens	300 µg	[96]
BI1361849 (CV9202)	CureVac	Unmodified	Protamine	Intramuscular	Prostate Cancer	1920 µg	[97]
MRT5005	Translate Bio	Unmodified	Internal LNP	Nebulization	Cystic Fibrosis	SAD: 24 mg MAD: 16 mg	[37]

Although fully synthetic srRNA vectors have reached the clinic, our understanding of srRNA biology is still limited. For example, although several species and subspecies of alphaviruses exist in nature, every fully synthetic srRNA clinical candidate to date has been derived from Venezuelan equine encephalitis virus (VEEV) virus. This is in part due to historical reasons since VEEV replicons were the first to be used for proof-of-concept studies. Indeed, the first report of any fully synthetic RNA vaccine delivered in vivo in a LNP-based formulation was a VEEV replicon [23]. This early srRNA-LNP vaccine elicited strong immune responses and protection in challenge studies in a rodent RSV model equivalent to a VRP vaccine. VRP clinical candidates encoding proteins for vaccines or biotherapeutics were a bit more diverse with trials using vectors derived from three types of viruses: VEE, Sindbis, and Semliki Forest. Nevertheless, we believe there is room for disruptive innovation in the field by mining the diversity present in nature for novel vectors derived from non-VEEV alphaviruses.

The idea that a given virally derived vector is not suitable for every application is exemplified by traditional viral vectors such as AAV. AAV is known to be a superior viral vector for gene therapy due to its low immunogenicity, specifically in generating CD8+ T cell responses. This feature of AAV allows for long-term gene expression and protein production in the host. Several characteristics of AAV lead to its poor recognition by the host immune system. One example of this is that AAV cannot efficiently transduce dendritic cells (DC), professional antigen-presenting cells that are central to CD8+ T cell responses, due to poor vector uncoating in the endosome and accessibility to cellular proteases [24]. Interestingly, engineering of the AAV capsid to render it less stable promoted uncoating in DCs and led to increased immunogenicity to a model antigen, suggesting that AAV variants can serve as vaccine platforms [24]. This shows that interactions between viral vectors and host cellular mechanisms affect their utility for therapeutic applications.

More recently for vaccines, the SARS-CoV-2 antigen led to protective responses in two distinct adenoviral platforms but failed in a VSV-based vector [25, 26]. Although the clinical data for the VSV-based SARS-CoV-2 vaccine has not yet been published, clinical failure was attributed to poor generation of protective immune responses. Importantly, VSV has shown promise for other viral antigens such as Ebola in licensed vaccines [27, 28]. Thus, viral vectors are not equally well-suited for all clinical applications (vaccines vs. biotherapeutics expression). Furthermore, not all viral vector-based vaccines will lead to protective immune responses for all antigens. Similarly, for srRNA-based approaches, the prototypical VEEV replicon has shown promise in the clinic in certain vaccines. However, like other viral vectors described above, we cannot assume that VEEV will serve as a plug-and-play vector for every antigen target, suggesting a need to expand a srRNA toolbox for additional vector backbones.

In addition to their utility for vaccines, the ability of srRNA to express high amounts of proteins with longer kinetics of expression, suggest their promise as vectors for expressing cytokines, enzymes, antibodies and other biotherapeutic proteins. After a single in vivo administration, protein expression from a synthetic srRNA can be detected up to seven weeks [23], overcoming repeated dosing with protein or conventional mRNA-based approaches. Animal data has shown that a single local administration of a srRNA-encoded cytokine can lead to therapeutic levels of a bioactive cytokine and result in tumor growth inhibition in vivo [29]. However, since srRNAs are virally derived, it is postulated that they are inherently immunogenic and unsuitable for systemic protein delivery. Nevertheless, the *Alphavirus* genus is rich in diversity. Specific species, subspecies, and variants interact differently with host cells and pathways, which can affect in vivo protein expression and immunogenicity. Thus, it is possible to develop novel srRNA platforms, customized for specific clinical applications by mining for additional sources of vectors derived from new species and subspecies of alphaviruses.

In addition to immune sensing pathways, mechanisms used by alphaviruses that affect host cell interactions are described below.

## srRNA vector–host cell interactions

To establish safe and efficacious dosing of vector-expressed vaccines and biotherapeutics, it is critical to achieve the therapeutic threshold for antigen and protein expression, respectively. Several cellular pathways contribute to how efficiently a protein is produced in situ within host cells, such as design of the inserted gene of interest through engineering to increase its half-life and stability, or by targeting it to specific cellular pathways with leader sequences. However, specifically for virally derived vectors, including srRNAs, the vector backbone itself may directly affect cellular mechanisms, protein expression, and presentation to the immune system.

**Fig. 1:**
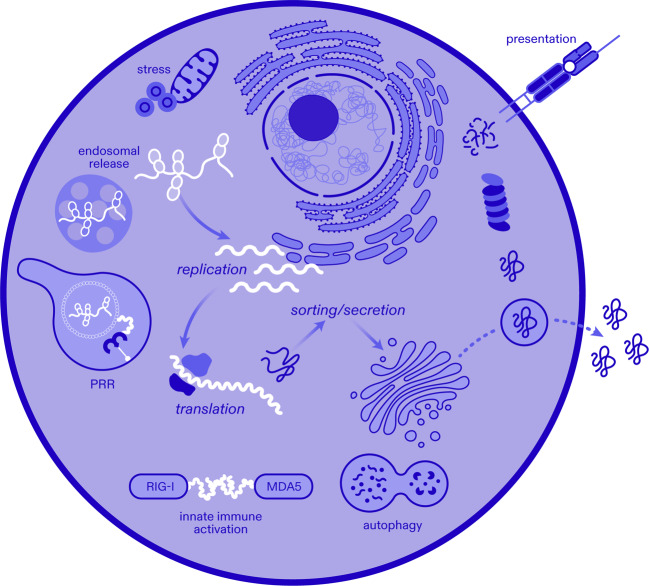
Self-replicating RNA and host cell interactions. Interplay of alphaviral srRNA vectors and host pathways are shown. Non-structural proteins and non-coding regions from diverse alphaviruses can differentially impact endosomal release, replication, translation, cellular stress, autophagy, innate immune activation through pattern recognition receptors (PRR) and sensors RIG-I/MDA5, protein sorting and secretion, as well as proteolytic processing and peptide presentation on major histocompatibility molecules for adaptive immune responses.

Of these pathways, presentation of proteins to T cells on MHC molecules is critical for vaccine-induced responses. This process relies on proteosomal degradation by the host cell machinery. To generate epitopes for CD8+ T cell responses, cytosolic and nuclear proteins are subjected to proteolytic degradation and ER transport for loading onto MHC class I molecules, which then traffics to the cell surface. CD4+ T cells recognize peptides bound to MHC class II molecules on the surface of professional antigen presenting cells, such as B cells, macrophages, and dendritic cells. Degradation of proteins for loading and surface presentation by MHC class II molecules occurs in the lysosomal compartment upon uptake of the targeted protein. Both MHC class I and class II pathways are regulated by various cellular pathways, some of which include interferon responses, autophagy, and cellular stress among others (Fig. 1).

### Downregulation of host gene expression

Beyond the essential activities of viral proteins that usurp cellular components to enable replication within a cell, viruses have evolved functions that have consequences to their host on an organismal level. Once replication initiates, alphaviruses efficiently shut off host gene expression by myriad mechanisms to suppress innate immune responses and delay detection by the host. While sequences in the structural proteins in most alphaviruses have been described to have these functions [30], there are redundant and additional activities of the nonstructural proteins which is divergent between species and subspecies. Numerous reports describe the suppression of host transcription and translation by non-structural protein (nsP) 2 of Old World (OW) alphaviruses (e.g. CHIKV, RRV, SFV, and SINV) by multiple mechanisms [31, 32], while these functions are primarily attributed to capsid protein from New World (NW) alphaviruses (e.g. VEEV, EEEV, and WEEV). In an example contrary to this dogma, expression of VEEV nsP2, but not EEEV nsp2, is sufficient to block host translation, although neither inhibit transcription, revealing that there are important differences between members within the historic OW/NW classifications [33]. Since srRNAs similarly induce activation of cellular antiviral sensors upon replication [34, 35], but lack structural proteins, it is important to consider the impact of the loss of structural protein functions and to harness properties of nonstructural genes that remain in the vector backbone.

### Type I interferon response

The innate immune system contains several microbial recognition molecules, such as pattern recognition receptors like Toll-like receptors located both at the cell surface and in endosomes, and cytosolic sensing molecules such as RIG-I, PKR, among others [36]. Triggering of these innate mechanisms by exogenous viral and nucleic acid vectors leads to induction of type I interferon responses. These responses can be seen as advantageous for vaccine development as IFNs can serve as adjuvants and elicit a more robust immune response by leading to maturation of APCs such as dendritic cells including enhanced antigen uptake and presentation. On the other hand, type I IFNs also modulate protein expression from viral and nucleic acid vectors by shutting down host protein translational mechanisms. Additionally, recent reports indicate that activation of the innate immune system may result in generation of anti-drug antibodies against encoded biologics [37]. For mRNA-based approaches, use of modified bases or improved manufacturing processes may reduce the host cell IFN response and innate immune activation [38]. However, viruses have evolved divergent immune evasion mechanisms that can subvert these host cellular pathways and may represent a more diverse and versatile solution. Furthermore, if each viral species and subspecies is recognized differently by the innate immune system, then each vector may need to be customized to the clinical application. For a given protein, some viral vectors will be better suited for pro-inflammatory uses, where additional IFN response are beneficial, whereas others may be advantaged for non-inflammatory expression of biotherapeutic proteins where durability and protein expression levels are more critical for efficacy.

### Viral virulence

Studies in model organisms provide valuable insights on unpredicted features between virulent and avirulent subspecies of alphaviruses. Virulence is a complex phenotype that can be derived from subtle differences in the way that a virus interacts with the immune system. Suthar et al. identified small dimorphisms in nsP1, nsP3, and E2 between the closely related avirulent SINV Girdwood and neurovirulent AR86 strains that are responsible for their pathogenesis [39]. Remarkably, the neurovirulence was gained or lost by swapping these sequences between the strains without affecting their establishment of infection in the brain and spinal cord or their replication in vitro. Further studies by Simmons et al. established that unlike Girdwood, a single mutation in nsP1 in AR86 enabled rapid and robust inhibition of STAT1/2 activation in response to IFN-γ and/or IFN-β, which resulted in decreased activation of Tyk2 and Jak1/2 [40]. In a second example, isolates of the 2006 breakout CHIKV DRDE-06 strain had as few as fifteen mutations from the prototype S27 strain, resulting in more severe clinical manifestations and in significantly faster replication in vitro [41], as well as an alteration in immune responses in macrophages [42]. As a further example, hematopoietic cell-specific microRNAs were identified in the 3’ UTR of the acutely virulent EEEV, which limits replication in myeloid cells and dramatically reduces the host’s protective immune response to infection [43]. This mechanism of immune evasion unique to EEEV and WEEV and the examples above emphasize the significance of small differences in sequences that may be overlooked in potential srRNA vectors that would otherwise be missed from in vitro experimentation. A recent in-depth review covered numerous known virulence determinants in the coding and non-coding regions of alphaviruses [44].

As new srRNAs are derived from alphaviruses it should be appreciated that the diversity of disease phenotypes and spread may provide insight on how the vector might behave in vivo. Modulation of affected pathways outlined above are known to impact inflammatory pathways in host cells and this mechanism of host cell regulation stands in contrast to the use of modified bases or alterations to the secondary structure used in mRNA approaches. Thus, when expressing a given therapeutic protein, we can tune the inflammatory environment by exploiting the divergent controls built into each srRNA vector without the need for secondary physical manipulations in manufactured drug products.

### Cellular stress and apoptosis

In addition to innate immune-based sensors, host cells respond to infection by developing a stress response or by regulated cell death (apoptosis). One feature of cellular stress is the formation of stress granules (SGs), defined as aggregates of protein-RNA complexes. SGs can accumulate following host translational arrest and serve to sequester viral RNA and prevent replication. As with other anti-viral host sensing mechanisms described above, some alphaviruses have evolved to modulate SG formation directly. For example, the conserved macrodomain of CHIKV nsP3 has been described to suppress SG formation and can drive SG disassembly [45]. While differences in SG formation have been observed between OW and NW alphavirus species, reduced/absent SGs in OW species may result in better overall expression of protein as they do not induce additional inflammation. However, it remains an open question whether SGs enhance or suppress immune response against a srRNA-encoded transgene. SGs may have a neutral or enhancing effect on immune responses in vaccine settings, and the disruption of their formation could result in a suppressed immune response against an encoded transgene.

Apoptosis is generally described to be a defense mechanism that limits virus replication and spread and plays a role in the induction of an immune response. Like most virus infections alphaviruses have been described to induce apoptosis, although its modulation varies between different species. The triggering of cell death has been described as early as alphavirus entry all the way through the late stage of infection. Notably, OW alphaviruses can act on cell-protective pathways, such as autophagy, which can delay apoptosis and promote viral spread. The relationship between alphaviruses and apoptosis has been recently reviewed here [46]. Since these activities have also been mapped to nsP, there is an expectation that new srRNAs derived from alphaviruses will have different transgene expression kinetics and immunostimulatory effects which may steer their utility between biotherapeutic vectors and vaccines.

### Autophagy

Autophagy is a homeostatic, regulated cellular process of lysosomal protein degradation and recycling that helps remove damaged components and organelles, leading to avoidance of cellular stress and death. In addition, autophagy plays a role in pathogen clearance and immune responses. As described previously for IFN responses, how viruses and their derived vectors can affect autophagy leads to differential protein expression and immunogenicity.

How autophagy impacts viral vector use is unclear. Some viruses encode molecules to promote autophagy to delay cell death that may result from cellular stress from viral infection and protein production, allowing the virus to remain and replicate within the host cell for longer durations. In therapeutic settings, this may lead to either high levels of protein expression in individual cells, or a longer durability of protein expression.

Conversely, autophagy can lead to better host cell immune responses by promoting antigen presentation, specifically for lysosome-derived MHC class II ligands [47]. Specifically, this has been demonstrated with the BCG vaccine for *Mycobacterium tuberculosis*, where promotion of autophagy mechanisms can bolster immune responses and vaccine efficacy [48, 49]. Even among OW alphaviruses there are differences in modulation of autophagy. It has been observed that CHIKV induces autophagy over the course of infection by inhibition of mTORC1 due to increased intracellular ROS and NO from oxidative stress [50]. In this context, autophagy is thought to be protective against apoptosis to promote CHIKV replication [50], although the consequences in vivo have been challenging to deconvolute since CHIKV nsP2 does not interact with murine NDP52 [51] and pharmacological induction and inhibition of autophagy have both improved clinical outcomes [50, 52]. In contrast, while there is also an elevated number of autophagosomes in SFV infection, it is thought to be instead through a mechanism of the expression of viral glycoproteins that block their degradation [53].

Thus, viral vectors may be advantaged as vaccines which encode promoters of autophagy, with the caveat that the autophagosomes retain their normal activity. Conversely, vectors that do not or negatively modulate autophagy may be better for expression of therapeutic proteins where immunostimulatory effects are undesirable.

## Delivery–host cell interactions

Deletion of the structural proteins in the alphavirus genome in synthetic srRNA vectors necessitates a formulation for optimal delivery in vivo. Although some preclinical examples of naked srRNA exist in the literature [6, 54], clinical delivery of srRNA vaccines has included a non-viral delivery system, such as LNPs or polymers [55].

Formulation can serve several purposes. It can protect the RNA vector from enzymatic degradation, neutralize the negative charge of the RNA to facilitate cellular uptake, and allow for endosomal escape to deliver the RNA molecule to cytoplasm.

### Lipid nanoparticles increase delivery efficiency and drive inflammatory responses

LNPs are most commonly used non-viral delivery system for srRNAs [5]. LNPs are made up of four key components that can affect their biophysical properties: an ionizable or cationic lipid, a helper lipid, cholesterol and PEG-lipid. Ionizable cationic lipids are only protonated in acidic environments, and this positive charge allows complexation with negatively charged RNA cargo. Patisiran [56], the only FDA-approved LNP-RNA therapeutic prior to the approved COVID-19 mRNA vaccines, showed how these particles could be produced at an industrial scale using an ethanol dilution process. Early prototype RNA-LNP vaccine formulations with ionizable cationic lipids such as DLinDMA utilized this scalable process and resulted in increased protein expression and improved immune responses compared to naked srRNAs, allowing reduction in administered dose [23, 55]. Further improvements in the design of optimized biodegradable ionizable lipids have resulted in improved potency of RNA vaccines in general [57].

LNPs are endocytosed by different pathways: micropinocytosis, phagocytosis, and clathrin-mediated and caveolae-mediated endocytosis [58]. Depending on the LNP composition, most ionizable lipid-based formulations have been identified from screening for liver delivery and require coating by ApoE to transfect cells in vitro, and presumably, in vivo receptor-mediated endocytosis [59, 60]. Although they lack true tissue tropism, preferential tissue targeting and certain biodistribution profiles can be achieved by either directly engineering targeting motifs, route of administration (ROA), or altering the composition and physicochemical characteristics of the particles [57, 61, 62].

Foremost, biodistribution of LNPs affects the host cell response and can be influenced by the ROA. For example, intravenous administration leads to high localization of LNPs in the liver and uptake by hepatocytes and Kupffer cells, while intramuscular administration leads to local protein expression in the muscle and draining lymph node, mainly in professional antigen presenting cells such as macrophages and B cells. The ROA can directly impact responses to the srRNA-LNP. For instance, the liver may be an ideal tissue for high level expression of biotherapeutic proteins by hepatocytes. However, its known tolerogenic environment is not ideal for production of antigenic proteins [63]. Thus, ROA can significantly impact in vivo responses to srRNAs.

Beyond ROA, size, particle surface charge and PEG-lipid structure of the LNP can further determine biodistribution and cellular tropism [62]. Specifically, size can impact extravasation of particles from blood vessels into tissues, with additional hurdles for tissues such as the lungs or entry into lymphatic vessels, where smaller particles can more easily move across this cellular barrier than larger ones [64–66]. However, larger particles are preferentially phagocytosed by APCs and can positively impact immunogenicity of RNA-LNP vaccines [64, 67]. LNP particle charge can be dictated by the cationic or ionizable lipid. The advantage of ionizable to cationic lipids is that they are only protonated in acidic environments, such as in endosomes, and neutrally charged while in circulation, allowing for increased circulation time in the host. Conversely, positively charged particles can interact more efficiently with cell surfaces, and possibly lead to better cellular uptake in first pass tissues such as the lung or liver [68].

Lastly, as mentioned previously, LNP formulations can also affect immune pathways directly [69–71], for example by binding of the lipids themselves to pattern recognition receptors. This may be beneficial for vaccines as it can adjuvant the immune responses, but unwanted for expression of biotherapeutics where it can lead to anti-drug antibodies and premature clearance of protein expression. Studies have shown that some LNPs directly synergize with srRNA to generate proinflammatory programs that drive immune responses against encoded proteins [69]. Thus, while LNP-formulated medicines exist for gene therapy indications, some toxicities have been reported with chronic use [71–76]. One potential way to circumvent immune activation by LNPs is to increase the PEG content [77] or addition of stealth polypeptides, such as polysarcosine [78], to shield the particle from the immune system. Additional scanning of lipid libraries to find immune stealth candidates has also led to newer LNP compositions that can deliver nucleic acid cargos, but may not lead to immune cell activation [72]. Nevertheless, such compositions have not been advanced clinically in combination with fully synthetic srRNA vectors.

### Polymers may drive efficient protein expression without inducing robust immune responses

However, as for the viral vectors described above, not all formulations behave similarly even with the same payload (srRNA and encoded protein). Specifically, LNP formulations have been categorically described as proinflammatory and advantaged for use in vaccines, where additional inflammation is beneficial to generate immune responses [69]. Additionally, cationic nanoemulsions and polymer formulations have also been validated for srRNA-based vaccines [6, 55, 79, 80]. Interestingly, however, a recent head-to-head comparison of the same srRNA payload formulated in either an LNP or a bioreducible polymer pABOL formulation showed that, although higher levels of protein were detected from polymer-formulated srRNA, poor immune responses were detected in vivo [81]. The lower immunogenicity of pABOL was associated with lack IL-6 induction, a cytokine with a known role in CD4 + T cell responses and humoral immunity [82–84]. These preliminary data suggest that delivery strategies need to be adapted to the delivered protein, and in cases where additional immune stimulation is unwanted, polymers are a suitable option for synthetic srRNA delivery.

Thus, similar to what was described for the srRNA vector itself, due to the diverse trafficking, tropism, and immunogenicity, the formulation should itself be considered an independent variable in the final drug product.

## Concluding remarks

Recent scientific advances have revealed the promise of fully synthetic srRNA products in oncology and infectious disease, but also highlighted the many variables complicating their development. Mining the diversity of vectors present in nature, combining with formulations fitting the purpose of each product, and eschewing a one-size-fits-all approach are key ingredients in the successful development of future srRNA products. Optimal drug product design will require regarding the biotherapeutic protein or antigen, vector, and delivery combination as fully independent variables requiring empirical determination. By leveraging this deliberative approach, we will collectively accelerate the time to market for srRNA-based products and deliver on srRNAs promise.
